# Unifocalization of Major Aortopulmonary Collateral Arteries (MAPCAs) and Native Pulmonary Arteries in Infancy—Application of 3D Printing and Virtual Reality

**DOI:** 10.3390/jcdd11120403

**Published:** 2024-12-13

**Authors:** Jacek Kolcz, Anna Rudek-Budzynska, Krzysztof Grandys

**Affiliations:** 1 Jagiellonian University, Collegium Medicum, Department of Pediatric Cardiac Surgery, 31-007 Krakow, Poland; 2 Department of Anesthesiology, University Children’s Hospital, 30-663 Krakow, Poland

**Keywords:** major aortopulmonary collateral artery, unifocalization, congenital heart defect

## Abstract

Background. Major aortopulmonary collateral arteries (MAPCAs) are rare remnants of pulmonary circulation embryological development usually associated with complex congenital anomalies of the right ventricular outflow tract and pulmonary arteries. Effective management requires surgical unifocalization of MAPCAs and native pulmonary arteries (NPAs). Traditional imaging may lack the spatial clarity needed for precise surgical planning. Aim. This study evaluated the feasibility of integrating three-dimensional (3D) printing and virtual reality (VR) into preoperative planning to improve surgical precision, team communication, and parental understanding. In a prospective cohort study, nine infants undergoing MAPCA unifocalization were included. Four patients underwent conventional imaging-based planning (control), while five were additionally assessed using VR and 3D-printed models (intervention). The outcomes measured included operative times, team confidence, collaboration, and parental satisfaction. Statistical analysis was performed using standard tests. Results. The intervention group had shorter operative and cardiopulmonary bypass times compared to the control group. Intraoperative complications were absent in the VR/3D group but occurred in the control group. Medical staff in the VR/3D group reported significantly improved understanding of anatomy, surgical preparedness, and team collaboration (*p* < 0.05). Parents also expressed higher satisfaction, with better comprehension of their child’s anatomy and surgical plan. Conclusions. VR and 3D printing enhanced preoperative planning, surgical precision, and communication, proving valuable for complex congenital heart surgery. These technologies offer promising potential to improve clinical outcomes and patient–family experiences, meriting further investigation in larger studies.

## 1. Introduction

Major aortopulmonary collateral arteries (MAPCAs) are rare entities frequently associated with congenital heart defects characterized by severe multilevel stenosis or atresia of the right ventricular outflow tract (RVOT), pulmonary valve abnormalities, and hypoplasia of the main pulmonary artery and its branches [1]. These complex conditions exhibit significant variability in pulmonary blood flow’s origins and supply routes. MAPCAs originate from the aorta or its primary branches and connect distally to the pulmonary arterial system, providing crucial blood supply to the lungs [2]. Depending on the degree of underdevelopment of the native pulmonary vessels, segments of the lungs may be supplied exclusively by MAPCAs or by a dual system involving both MAPCAs and native pulmonary arteries (NPAs) [3].

The surgical management of MAPCAs aims to achieve biventricular circulation, wherein unifocalized MAPCAs and NPAs jointly support pulmonary blood flow. Given the heterogeneity in the development of native pulmonary vessels and the anatomical variability of MAPCAs, surgical treatment often requires a staged approach. This typically involves establishing a systemic-to-pulmonary shunt with unifocalized pulmonary vessels or creating a right ventricular-to-pulmonary connection to promote adequate growth and development of the pulmonary vasculature [4,5].

Virtual reality (VR) and three-dimensional (3D) printing technologies have emerged as valuable tools that address the inherent limitations of conventional two-dimensional (2D) imaging modalities, allowing for more personalized and precise treatment planning. VR provides an immersive experience with genuine depth perception and the ability to manipulate anatomical models, facilitating enhanced surgical assessments and virtual training. Three-dimensionally printed models offer real-scale anatomical representations, improving communication among surgical teams and enhancing education for both medical professionals and patients’ families [6,7].

This study aims to assess the technical feasibility, clinical benefits, and user experience of utilizing 3D virtual reality (VR) and 3D printing technologies in the preoperative planning for surgeries in patients with rare and complex congenital heart defects involving MAPCAs unifocalization. Additionally, the effectiveness of communication with both the medical team and the patients’ parents was evaluated.

## 2. Materials and Methods

### 2.1. Study Design

This prospective non-randomized comparative cohort study was conducted at the University Children’s Hospital to evaluate the impact of integrating 3D printing and virtual reality (VR) technologies in preoperative planning for patients undergoing MAPCAs unifocalization. Outcomes were compared between two groups: a control group assessed with traditional imaging methods (e.g., echocardiography, computed tomography, or magnetic resonance imaging) and an intervention group that additionally utilized VR and 3D printing. During heart team discussions, patient selection for 3D printing and VR implementation was primarily guided by resource availability and clinical prioritization. These decisions were influenced by intermittent software licensing, financial constraints associated with 3D printing, and the complexity of anatomical presentations. The selection process aimed to include patients with comparable levels of defect complexity in both the intervention and control groups, ensuring a balanced comparison of outcomes. 

### 2.2. Study Population

Nine patients with complex congenital cardiac anomalies and MAPCAs undergoing unifocalization and corrective surgery between 2017 and 2023 were included. The control group (n = 4) received conventional diagnostic assessment, while the intervention group (n = 5) received VR and 3D printing-assisted planning. The median age at surgery was 213 days (29–306 days) for the VR/3D group and 220 days (35–340 days) for the control group. Preoperative imaging data, including computed tomography angiography (CTA) and catheterization angiography (CA), were available for all patients.

### 2.3. Inclusion and Exclusion Criteria

Inclusion criteria: (1) infants diagnosed with congenital heart defects with MAPCAs-dependent pulmonary circulation, (2) candidates for primary unifocalization of MAPCAs and NPAs, (3) availability of adequate imaging data, and (4) informed parental consent. Exclusion criteria included (1) incomplete clinical data, (2) insufficient imaging quality, (3) emergent surgery that precluded preparation of 3D models or VR simulations, and (4) technical infeasibility for using 3D printing or VR.

Case selection for 3D printing and VR was determined by the heart team, prioritizing complex anatomical presentations and cases where enhanced visualization was expected to influence surgical planning. Resource limitations (e.g., restricted software licenses) also influenced patient selection.

### 2.4. Data Collection and Outcome Measures

Baseline demographics, clinical history, and imaging data were collected for both groups. Intraoperative findings, surgical outcomes, hospital stay length, and postoperative complications were recorded prospectively. Additional data on the preparation and application of 3D models and VR were documented for the intervention group.

### 2.5. Survey Assessments

Parental comprehension and satisfaction were assessed post-surgery via a questionnaire. The survey included 10 statements across five domains: understanding of the child’s condition, surgical plan comprehension, communication clarity, anxiety reduction, and overall satisfaction with care. Responses were rated on a 5-point Likert scale (1 = Strongly Disagree, 5 = Strongly Agree).

Medical staff from both surgical and cardiology teams completed surveys for each case. Surveys were conducted primarily after the heart team’s qualification discussions, allowing physicians to evaluate the utility of the diagnostic tools before surgery. These evaluated the diagnostic method’s utility in surgical planning, confidence in the approach, and perceived impact on outcomes. The survey also contained 10 statements covering anatomy understanding, surgical preparation, intraoperative confidence, team communication, and satisfaction with VR and 3D printing. Responses were similarly rated on a 5-point Likert scale.

### 2.6. Three-Dimensional Printing for Personalized Anatomical Models

**Figure 1:**
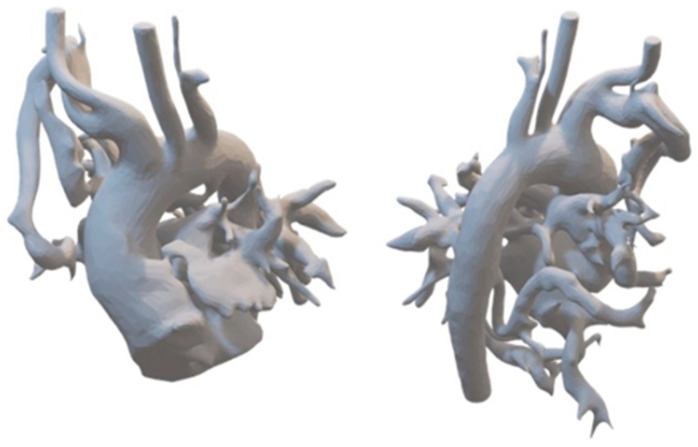
An example of a 3D-printed model of a patient with tetralogy of Fallot and multiple major aortopulmonary collateral arteries (MAPCAs). A prominent circumflex vessel is visible, originating from the right subclavian artery, along with MAPCAs branching from the descending aorta.

Patient-specific CT data saved in DICOM format were segmented using 3D Slicer software, with further refinements for stabilizing elements and design adjustments. Final files compatible with 3D printing were generated using FDM printing techniques (Orca Slycer) and printed using high-resolution 3D printing technologies (Figure 1).

### 2.7. Virtual Reality for Immersive Simulation

**Figure 2:**
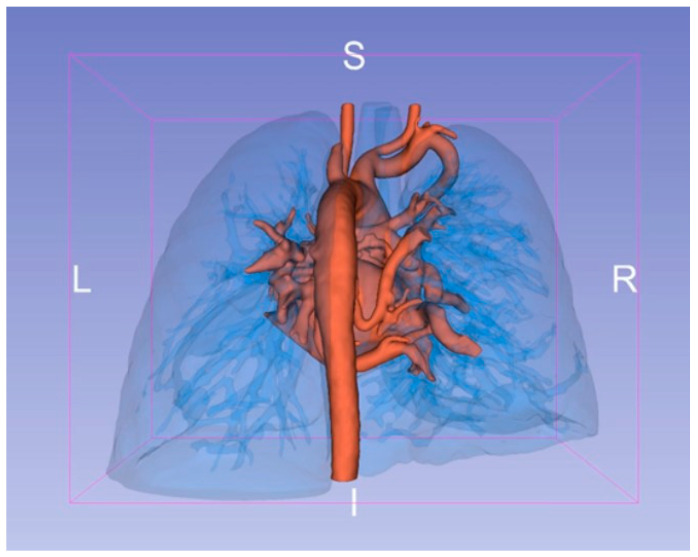
Three-dimensional reconstruction of the heart and lungs showcasing the topography of all detected MAPCAs. This model provides a comprehensive view of the vascular pathways, clearly delineating the origins, routes, and connections of each MAPCA with the native pulmonary arteries, offering a precise anatomical map to aid in preoperative planning and intraoperative guidance. L: Refers to “Left”; I: Refers to “Inferior”; R: Refers to “Right”; S: Refers to “Superior”.

**Figure 3:**
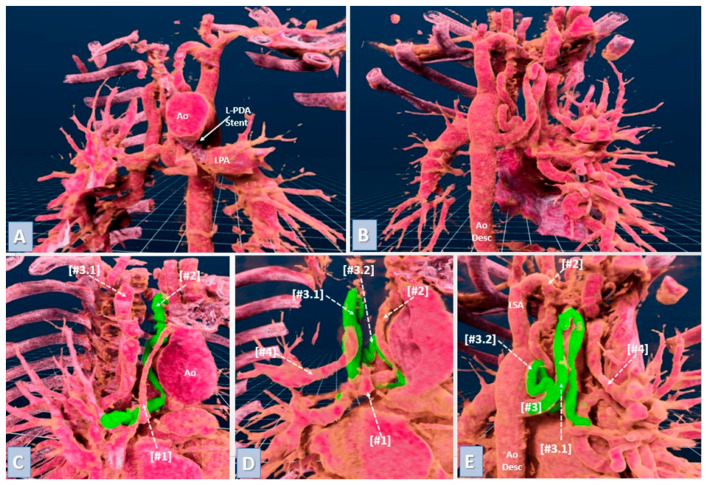
Virtual reality model, meticulously constructed from computed tomography images of a patient diagnosed with tetralogy of Fallot (TOF), pulmonary trunk agenesis, multiple MAPCAs, and non-confluent pulmonary arteries following a right-sided modified Blalock–Taussig shunt to the right pulmonary artery (RPA) and stenting of the arterial duct to supply the left pulmonary artery (LPA). (**A**): Anterior view showing the stented arterial duct that supplies the non-confluent LPA, a key anatomical feature providing crucial blood flow to the left lung; (**B**): Posterior view highlighting the MAPCAs originating from the descending aorta, which are primarily responsible for the blood supply to the right lung; (**C**): Anterior view detailing the blood supply to the right lung. [#1] Modified Blalock–Taussig shunt linking the RPA to the proximal segment of the brachiocephalic trunk. [#2] Right-sided MAPCA originating from the proximal left subclavian artery and connecting to the native RPA in the right lung hilum (marked in green). [#3.1] A partially visible MAPCA segment arising from the descending aorta; (**D**): Anterior view further illustrating the MAPCA from the descending aorta ([#3]), which divides proximally into two right-sided branches ([#3.1] and [#3.2]), supplying the same area as the native RPA. Additionally visible are the following: [#4] A distal segment of an R-MAPCA from the right subclavian artery to the right lung’s upper lobe. [#1] Partially visible native RPA. [#2] MAPCA from the left subclavian artery to the right lung. [#4] Distal segment of the MAPCA from the right subclavian artery to the right lung. (**E**): Posterior view displaying the MAPCA from the descending aorta ([#3]), dividing into branches ([#3.1] and [#3.2]) that converge at the right hilum, overlapping with the area supplied by the native RPA. Also seen are [#2] MAPCA originating from the left subclavian artery. [#4] Middle and distal segments of the MAPCA from the right subclavian artery. L—left, R—right, S—superior, I—inferior.

VR models were created from preoperative CTA scans in DICOM format using automated segmentation to enhance visualization of key structures like pulmonary artery branches and MAPCAs. These data were imported into VR software (VEA (visual exploratory activity) Simulations platform, http://vea-sim.com), creating an interactive 3D environment reviewed by an experienced physician to confirm anatomical accuracy (Figure 2). Surgeons interacted with the VR models using headsets and controllers, providing a fully immersive planning experience (Figure 3).

The computed tomography angiography (CTA) data segmentation for 3D printing included mapping critical structures such as the bronchial tree, esophagus, and MAPCAs. These models were fabricated to enable tactile exploration of their spatial relationships. During preoperative planning, surgeons assessed potential conflicts, such as compression of the bronchial or esophageal structures, by tracing MAPCA paths relative to these adjacent structures. In VR simulations, the anatomical data were rendered into immersive 3D environments, allowing the surgical team to rotate, magnify, and interact with the model. This dynamic visualization helped predict and mitigate complications by simulating planned vessel mobilization and anastomosis routes. For example, the proximity of MAPCAs to the left bronchus and esophagus was examined in VR, guiding adjustments to the planned trajectory for vessel relocation.

### 2.8. Statistical Analysis

Descriptive statistics included medians and interquartile ranges (IQR) for continuous not normally distributed variables or mean ± SD for normal distributions. The Mann–Whitney U test was used to compare continuous variables between the intervention and control groups, while Fisher’s exact test analyzed categorical variables. The effect size for the difference in operative and cardiopulmonary bypass times, as well as intensive care unit recovery time and hospitalization time between the intervention and control groups, was evaluated using Cliff’s delta and a permutation test to confirm statistical significance. Statistical significance was set at *p* < 0.05, and all analyses were performed using Statistica 13 software.

## 3. Results

### 3.1. Enhanced Surgical Precision and Reduced Operative Time

All patients in both VR/3D printing and control groups underwent midline sternotomy and primary unifocalization of all MAPCAs and native pulmonary arteries with cardiopulmonary bypass. Cardioplegic cardiac arrest was performed to facilitate right ventriculotomy, VSD assessment, and RV–PA conduit implantation. During the procedure, MAPCAs were carefully mobilized and gathered in the middle inferior mediastinum. In two cases, a large MAPCA was routed through the mediastinal pleura to anastomose end-to-end with a native pulmonary artery or a reconstructed neo-pulmonary trunk. Most MAPCAs were repositioned and anastomosed end-to-side or side-to-side with properly cut hypoplastic pulmonary branches. Narrow MAPCAs were widened with a pulmonary homograft patch to optimize blood flow. After completing the anastomoses, the neo-pulmonary trunk was reconstructed with a homograft, and the right ventricular outflow tract was connected using a reinforced PTFE vessel or a valved RV–PA conduit tailored to the patient size.

The integration of 3D printing and VR significantly reduced operative and bypass times as well as ICU time. The intervention group demonstrated a shorter operative time (6.5 h versus 8.3 h) with Cliff’s delta calculated at −0.88, indicating a large effect size favoring the intervention. The permutation test yielded a statistically significant difference with a *p*-value of *p* = 0.022. Similarly, a reduction in cardiopulmonary bypass time was observed in the intervention group (126 min versus 158 min), with Cliff’s delta of −0.84, reflecting a large effect size. The permutation test result was significant, with a *p*-value of *p* = 0.03. The intervention group experienced shorter postoperative intensive care time, with a Cliff’s delta value of −0.84, also representing a large effect size. The permutation test yielded a *p*-value of *p* = 0.039, indicating statistical significance (Table 1).

### 3.2. Educational Impact on Surgical Teams and Families

The use of VR and 3D printing offered significant educational benefits, enhancing team understanding of patient-specific anatomy and preparation for surgery. Junior team members, in particular, gained familiarity with complex cardiovascular structures through 3D models, improving their ability to assist effectively during surgery. This preparation translated into better teamwork, reduced uncertainty, and a more coordinated intraoperative environment (Table 2).

In the VR/3D group, medical staff rated their understanding of patient-specific anatomy significantly higher (Mean ± SD: 4.75 ± 0.45) than in the control group (4.0 ± 0.75, *p* = 0.0318). Surgical preparation scores were also higher in the intervention group (4.6 ± 0.6 vs. 4.0 ± 0.9, *p* = 0.0495), and team communication and collaboration showed improvement with VR/3D use (4.75 ± 0.5 vs. 4.2 ± 0.8, *p* = 0.0318). The highest-rated factor in the VR/3D group was satisfaction with the technology in surgical planning (4.85 ± 0.35 vs. 4.1 ± 0.7, *p* = 0.0260), with trainees rating VR and 3D printing particularly positively, reflecting these technologies’ support for those in training (Table 2).

The 3D-printed models and VR simulations also significantly improved communication with families, as they facilitated clearer explanations of congenital defects and surgical plans. Parents in the VR/3D group rated their understanding of their child’s anatomy higher (4.7 ± 0.45) than those in the control group (3.9 ± 0.85, *p* = 0.0245). Additionally, parents’ confidence in expected surgical outcomes and overall satisfaction were greater in the VR/3D group (4.9 ± 0.3 vs. 4.3 ± 0.6, *p* = 0.0215), indicating improved trust and reduced preoperative anxiety (Table 3).

Moreover, survey response analysis revealed distinct advantages of VR and 3D printing for different aspects of preoperative planning. Junior team members reported greater improvement in intraoperative confidence and understanding of surgical pathways with VR (Mean ± SD: 4.8 ± 0.4). In contrast, senior staff valued 3D-printed models more for their ability to visualize and plan precise anastomoses, particularly in cases with multiple MAPCAs and non-confluent pulmonary arteries (Mean ± SD: 4.9 ± 0.3). Parental responses also varied; while the 3D-printed models were highly effective for conveying detailed anatomical structures, VR better illustrated the overall spatial complexity of the procedure.

### 3.3. Intraoperative Guidance and Decision Making

The availability of 3D-printed models during surgery provided a tangible reference for real-time decision making, particularly in complex anatomical situations. These models enabled precise scaling and orientation, aiding the surgical team in performing accurate anastomoses. Combined with VR simulations, this real-time guidance helped surgeons navigate complex anatomy with confidence, contributing to successful outcomes in all VR/3D cases. Feedback indicated increased intraoperative confidence with new technologies (4.5 ± 0.5 vs. 3.8 ± 1.1).

The use of 3D printing and VR significantly enhanced the ability to evaluate spatial relationships between MAPCAs, bronchial structures, and the esophagus, which are critical for planning unifocalization surgery. For instance, a 3D-printed model revealed a MAPCA originating from the descending aorta that coursed behind the left main bronchus and was in close proximity to the esophagus. This allowed surgeons to plan a mobilization path that minimized the risk of bronchial compression and esophageal injury during vessel relocation and anastomosis. Similarly, VR simulations provided dynamic visualization of these relationships, allowing the surgical team to rehearse approaches in complex cases. When VR revealed a large MAPCA closely related to the right main bronchus, it enabled precise surgical handling, preventing potential airway obstruction. Both technologies provided complementary and indispensable insights, contributing to successful unifocalization without postoperative airway or esophageal complications.

In summary, integrating VR and 3D printing technologies demonstrated substantial benefits in surgical precision, reduced operative and bypass times, enhanced teamwork, and improved communication with families. These results underscore the potential for VR and 3D printing to transform surgical planning and education, supporting their broader application in complex congenital surgeries involving MAPCAs.

## 4. Discussion

The integration of 3D printing and virtual reality (VR) technologies marks a significant advancement in the management of complex congenital heart defects involving major aortopulmonary collateral arteries (MAPCAs) [8]. Traditional imaging methods like 2D echocardiography and computed tomography angiography (CTA) often lack the spatial details necessary for intricate preoperative planning, limiting a comprehensive understanding of three-dimensional (3D) cardiovascular structures critical for precise surgical decision making [9]. This study, in line with emerging evidence, underscores the transformative potential of 3D printing and VR in enhancing surgical approaches to MAPCAs unifocalization.

Recent studies have highlighted that 3D printing can address some of these imaging limitations by creating detailed tactile models that provide a more nuanced view of spatial relationships in cardiac structures [10,11]. For instance, the use of 3D-printed heart models has demonstrated a significant impact on surgical planning, enabling surgeons to develop individualized strategies that align with specific anatomical challenges in patients with CHD [12]. Our findings align with these studies; we found that 3D-printed models allowed surgeons to interact directly with patient-specific anatomy, offering crucial insights into the optimal arrangement and anastomosis of MAPCAs and native pulmonary arteries. This hands-on experience proved invaluable for accurate preoperative planning in complex cases, further supporting the established evidence that 3D printing can effectively enhance surgical strategy formulation [13,14].

Similarly, VR offers a distinct advantage over traditional 2D visualization by providing an immersive 3D interactive experience that benefits surgeons and trainees alike. Prior studies have demonstrated VR’s role in enhancing diagnostic assessment accuracy and its utility as a collaborative tool for multidisciplinary teams [15,16]. Our findings reinforce these benefits, as VR significantly improved the surgical team’s understanding of complex cardiac anatomy, facilitating both decision making and real-time intraoperative navigation. In alignment with earlier studies [6], VR, in our cohort, enabled surgeons to rehearse procedures virtually, which likely contributed to the increased surgical confidence and reduced operative times observed. The ability to practice different approaches in a safe virtual environment has been previously correlated with improved intraoperative confidence, an effect observed in our study as reflected in high ratings from surgical staff.

One of the most valuable contributions of 3D printing and VR in this study was their ability to depict the anatomical relationships between MAPCAs, the bronchial tree, and the esophagus in unprecedented detail. This enhanced spatial understanding was critical for surgical planning, particularly in cases with complex vascular configurations. Three-dimensionally printed models allowed surgeons to identify potential bronchial or esophageal compression risks and develop tailored vessel mobilization strategies. VR provided complementary benefits, enabling dynamic exploration of these relationships and facilitating virtual rehearsals of surgical maneuvers. The capacity to simulate vessel relocation pathways in VR helped minimize the risk of complications, such as airway obstruction or esophageal injury, which are common concerns in unifocalization surgeries.

The innovative roles of 3D printing and VR in congenital heart disease surgeries have been documented by other researchers, who observed that both technologies offer additional value to cardiac surgeons and cardiologists by enhancing spatial visualization beyond the scope of traditional 2D imaging [17]. Beyond congenital heart disease surgery, these technologies have also shown significant potential in prenatal diagnosis, enabling improved visualization and understanding of fetal cardiac anomalies. Monteiro Pereira Leite et al. demonstrated how 3D printing and virtual modeling of a fetal left ventricular giant aneurysm facilitated an accurate prenatal diagnosis and enhanced multidisciplinary discussions for planning postnatal interventions [18]. Similarly, Bravo-Valenzuela et al. highlighted the utility of virtual reality in navigating a normal fetal heart, illustrating its educational and diagnostic value in complex prenatal scenarios [19]. These applications underscore the broader utility of 3D printing and VR not only in surgical contexts but also in aiding early and precise fetal cardiac assessments [17]. Specifically, our findings indicated that 3D printing provided a tangible model for studying static anatomical structures, while VR allowed dynamic immersive simulations that aided in surgical planning. These complementary roles were particularly evident in their impact on team communication and collaboration, as VR enabled a shared virtual exploration of complex anatomical structures. This cohesive teamwork benefit aligns with studies by Lau et al. [7] and Chessa et al. [18], both of whom highlighted the potential of VR to improve intraoperative coordination. Moreover, the combined data from staff and parental surveys illustrated the added educational and communicative value of these technologies. In the VR/3D group, the surgical team reported higher confidence in their understanding of anatomy, surgical preparation, and communication (Table 2), resulting in better operative and bypass times and shorter ICU time (Table 1). Parents in the VR/3D group also demonstrated improved comprehension of their child’s anatomy and procedure, which likely contributed to reduced preoperative anxiety and greater satisfaction with care (Table 3).

### 4.1. Educational Impact and Team Collaboration

One of VR’s most significant contributions to surgical education lies in its immersive, interactive, and cost-effective nature. Multiple studies [19,20] have reported VR’s potential in surgical training, particularly in facilitating skill acquisition through repetitive practice in a risk-free setting. Such training can address the limitations of traditional “see one, do one, teach one” approaches, helping to reduce medical errors and enhance patient safety. Our study demonstrated VR’s effectiveness in educating junior staff, who used VR and 3D models to familiarize themselves with complex cardiovascular structures. VR’s immersive quality also enabled improved team cohesion, allowing all members to share a unified understanding of the surgical approach, reducing uncertainty and enhancing coordination. This finding resonates with prior studies that showed VR’s potential to foster teamwork and build confidence through hands-on virtual practice [21,22,23].

Furthermore, our study highlighted the educational and communication benefits of VR and 3D printing for the families of patients. Research by Zhao et al. [11] and Yoo et al. [9] has shown that both technologies aid in explaining complex procedures to families, fostering trust and understanding. In our cohort, parents in the VR/3D group reported a higher level of understanding and satisfaction, likely due to the clearer explanations these models provided. This aligns with the growing recognition of VR and 3D printing as valuable tools for family-centered care, supporting both patient education and family engagement in the treatment process.

### 4.2. Complementary Roles of 3D Printing and VR

While 3D printing and VR offer unique advantages, our study underscores that they are most effective when used in tandem. Three-dimensionally printed models provided a physical reference for understanding structural anatomy, which is particularly useful for studying static spatial relationships. In contrast, VR’s adaptability and interactivity made it ideal for simulating complex maneuvers, offering a dynamic preview of challenging surgical procedures. This dual approach supports comprehensive preoperative assessments and reinforced anatomical understanding, illustrating that 3D printing and VR are complementary, not interchangeable, in CHD surgery.

While 3D printing and VR are often grouped, their roles in surgical preparation and education are distinct and complementary. Three-dimensional printing provided tactile physical models that were instrumental for understanding static spatial relationships and detailed anatomical structures, such as the MAPCAs and native pulmonary arteries. This was particularly beneficial during surgical planning and intraoperative decision making, where a tangible reference proved invaluable. Conversely, VR offered an immersive and dynamic environment, allowing surgeons to simulate surgical procedures and practice different approaches, which was particularly helpful for junior team members and intraoperative preparedness. Feedback from the surveys supports this differentiation. Junior members rated VR more favorably due to its interactive nature, while staff members found 3D-printed models critical for grasping the anatomical details. Parental understanding and satisfaction were equally enhanced by both tools, but for different reasons: 3D models provided a hands-on explanation of anatomy, while VR made complex spatial relationships more comprehensible. These findings highlight the complementary, rather than interchangeable, roles of 3D printing and VR in preoperative planning and communication.

### 4.3. Limitations and Future Directions

Despite promising results, our study is limited by its small sample size, tied to the preliminary nature of introducing these technologies with restricted accessibility. This limitation constrains the generalizability of our findings, and further research with larger patient cohorts is needed to validate these observations. We also acknowledge potential biases arising from the non-randomized selection of patients based on resource availability, the timing of surveys, and satisfaction evaluations by surgical staff. While efforts were made to minimize bias through consistent operative techniques and anonymous survey administration, the inherent limitations of this methodology must be recognized. Future studies should also prioritize developing standardized protocols for 3D printing and VR in CHD surgery to ensure consistent outcomes across clinical settings and improve cost-effectiveness.

In addition, expanding VR capabilities to simulate team dynamics through multi-user platforms could further enhance collaborative practice. Broadening these technologies’ applications into postoperative care, patient education, and long-term follow-up would deepen their utility in managing complex congenital heart defects. Moreover, integrating artificial intelligence (AI) and machine learning could improve the accuracy of simulations by tailoring them to each patient’s unique anatomy. As these advancements take shape, VR and 3D printing could play a pivotal role in training the next generation of cardiac surgeons, complementing traditional methods, reducing medical errors, and improving patient outcomes.

## 5. Conclusions

The implementation of 3D printing and VR technologies in preoperative planning for complex congenital heart surgeries involving MAPCAs demonstrated enhanced surgical precision, reduced operative and intensive care times, and improved educational experiences for surgical teams and families. These findings support the broader integration of VR and 3D printing as valuable tools in routine clinical practice, advancing personalized and precise treatment strategies for congenital heart conditions.

## Figures and Tables

**Table 1:** Clinical characteristics and outcomes of patients in the intervention (VR and 3D printing) and control (without VR and 3D printing) groups.

Parameter	Intervention Group (VR and 3D Printing)	Control Group (Without VR and 3D Printing)	*p*
Number of patients, n (%)	5 (55.6%)	4 (44.4%)	n/a
Age, days, median [IQR]	213 (9–306)	220 (35–340)	0.70
Complexity of MAPCAs (number of MAPCAs per patient)	4 (3–6)	4.5 (3–7)	0.82
Total operative time, hours, median (IQR)	6.5 (5.5–7.5)	8.3 (7.2–9.6)	0.023
Cardiopulmonary bypass time, minutes, median (IQR)	126 (110–145)	158 (132–176)	0.030
Intraoperative complications	None	Bleeding in 2 cases, delayed sternal closure (2 patients)	n/a
Postoperative ICU recovery time, days, median (IQR)	7.0 (5.0–9.0)	10.0 (8.0–14.0)	0.039
Total hospital stay, days, median (IQR)	14.0 (12.0–18.0)	18.0 (16.0–22.0)	0.057

Abbreviations: IQR, interquartile range; VR, virtual reality, MAPCA, major aortopulmonary collateral artery; ICU, intensive care unit; n/a, not applicable.

**Table 2:** Comparison of medical staff evaluations on surgical planning with and without VR and 3D printing technologies.

Domain	Question	With VR and 3D Printing (Mean ± SD/Median [IQR])	Without VR and 3D Printing (Mean ± SD/Median [IQR])	*p*-Value
Understanding of Anatomy	Q1: Understanding of patient-specific anatomy	4.75 ± 0.45/5.0 [1.0]	4.0 ± 0.75/4.0 [1.5]	0.0318
Preparedness for Surgery	Q2: Surgical preparation	4.6 ± 0.6/5.0 [1.0]	4.0 ± 0.9/4.0 [1.5]	0.0495
Intraoperative Confidence	Q3: Intraoperative confidence	4.5 ± 0.5/4.0 [1.5]	3.8 ± 1.1/4.0 [2.0]	0.1320
Ease of Communication	Q4: Team communication and collaboration	4.75 ± 0.5/5.0 [0]	4.2 ± 0.8/4.0 [1.0]	0.0318
Overall Satisfaction	Q5: Overall satisfaction with technology in surgical planning	4.85 ± 0.35/5.0 [1.0]	4.1 ± 0.7/4.0 [1.0]	0.0260
Specialist Ratings	Overall rating from specialists	4.5 ± 0.5	4.2 ± 0.6	0.0578
Trainee Ratings	Overall rating from trainees	4.8 ± 0.4	3.7 ± 0.9	0.0369

Abbreviations: SD, standard deviation; IQR, interquartile range; VR, virtual reality.

**Table 3:** Comparison of parental evaluations on surgical understanding and communication with and without VR and 3D printing technologies.

Domain	Question	With VR and 3D Printing (Mean ± SD/Median [IQR])	Without VR and 3D Printing (Mean ± SD/Median [IQR])	*p*-Value
Understanding of Anatomy	Q1: Understanding of child’s specific anatomy	4.7 ± 0.45/5.0 [1.0]	3.9 ± 0.85/4.0 [1.5]	0.024
Clarity of Procedure	Q2: Understanding of planned surgical procedure	4.8 ± 0.4/5.0 [0.5]	4.0 ± 0.9/4.0 [1.0]	0.032
Confidence in Outcome	Q3: Confidence in expected surgical outcome	4.6 ± 0.6/5.0 [1.0]	4.1 ± 1.0/4.0 [1.5]	0.058
Satisfaction with Communication	Q4: Facilitated communication with surgical team	4.85 ± 0.35/5.0 [1.0]	4.2 ± 0.7/4.0 [1.0]	0.028
Overall Satisfaction	Q5: Overall satisfaction with information provided about surgery	4.9 ± 0.3/5.0 [0]	4.3 ± 0.6/4.0 [1.0]	0.021

Abbreviations: SD, standard deviation; IQR, interquartile range; VR, virtual reality.

## Data Availability

Data available after requesting correspondence author.
